# Clinical characteristics of immune-related adverse events after immune checkpoint inhibitor therapy with or without chemotherapy in patients with antinuclear antibody-positive malignancies

**DOI:** 10.3389/fimmu.2026.1854574

**Published:** 2026-09-14

**Authors:** Hanghui Yu, Enyong Dai, Jianpeng Xing, Shengnan Liu, Huijie Xiao, Zhengjia Liu

**Affiliations:** 1 Second Ward of the Oncology Department, China–Japan Union Hospital of Jilin University, Changchun, Jilin, China; 2 Department of Gastrointestinal Colorectal Surgery,China-Japan Union Hospital of Jilin University, Changchun, Jilin, China; 3 Department of Breast Surgery, China-Japan Union Hospital of Jilin University, Changchun, Jilin, China; 4 Department of Thoracic Surgery, China-Japan Union Hospital of Jilin University, Changchun, Jilin, China

**Keywords:** acquired resistance, antinuclear antibody, autoantibodies, chemotherapy, drug resistance, immune-related adverse events, immunotherapy, tumor immune microenvironment

## Abstract

**Background:**

Antinuclear antibodies (ANA) are detected in cancer patients before ICI therapy, but their association with immune-related adverse events (irAEs) and outcomes is uncertain. This study aimed to characterize irAEs and tolerability in ANA-positive patients receiving ICI ± chemotherapy, and explore implications for resistance.

**Methods:**

We retrospectively analyzed 121 solid tumor patients who received ICI therapy ± chemotherapy at the China-Japan Union Hospital (2021-2026). By baseline ANA titer (≥1:80 positive), patients were divided into positive (n=60) and negative (n=61) groups. We compared the incidence, severity, onset time, organ distribution, glucocorticoid use, and immunotherapy outcomes of irAEs between the two groups.

**Results:**

The incidence of irAEs was significantly higher in the ANA-positive group than in the ANA-negative group (70.0% vs. 49.2%, OR = 2.41, 95% CI: 1.14–5.09, P = 0.026). A significant dose–response relationship was observed between ANA titer and irAE risk (P_trend = 0.036), and each increase in titer category was associated with an 80% increase in risk (OR = 1.80). Autoantibody burden associated positively with irAE incidence (0 positive: 54.84%; 1 positive: 72.73%; ≥2 positive: 83.33%), although irAE severity did not increase with antibody burden (P = 0.581). The ANA positive group showed a trend toward higher irAE rates across multiple organ systems, including endocrine, pulmonary, and hepatic toxicities. There was no significant difference in the proportion of patients who discontinued ICIs because of irAEs between the two groups (20.0% vs. 21.3%, P = 1.000), and glucocorticoid use was also similar (33.3% vs. 23.3%, P = 0.357). Notably, the ANA-positive group showed a lower proportion of discontinuation due to disease progression (21.6% vs. 34.4%, P = 0.157), suggesting a potential link to delayed chemoresistance.

**Conclusion:**

Baseline ANA positivity may be a potential predictor of irAE development after ICI therapy with or without chemotherapy and shows a clear dose effect relationship. ANA-positive patients exhibited a clinical pattern characterized by a high frequency but manageable irAEs; under standardized management, this did not compromise treatment continuity and may indicate stronger antitumor immune activation with potential survival benefit,potentially delaying acquired resistance to ICIs. Baseline autoantibody testing may help identify high-risk patients and guide resistance-overcoming monitoring strategies.

## 1 Introduction

Malignancies remain a major global health challenge because of their high incidence and mortality. The advent of immune checkpoint inhibitors (ICIs) has reshaped the traditional cancer treatment landscape. Their broad application in a variety of solid tumors has demonstrated substantial therapeutic efficacy. By relieving tumor-mediated suppression of the host immune system and reactivating endogenous antitumor immunity, ICIs, in combination with conventional chemotherapy and targeted therapy, have introduced a new paradigm for cancer treatment (1, 2).

However, the clinical issues associated with ICI therapy are becoming increasingly apparent (3). Immune checkpoint inhibitors, represented by programmed cell death protein 1 (PD-1)/programmed cell death ligand 1 (PD-L1) inhibitors, may excessively activate the immune system. T-cell dysregulation, autoantibody production, complement activation, increased cytokine release, intestinal dysbiosis, and even genetic factors (4) can disrupt immune tolerance, enhance immune reactivity, increase sensitivity to self-antigens, and ultimately lead to attacks on normal organs. This process may trigger inflammatory cytokine storms and a variety of toxicities collectively known as immune-related adverse events (irAEs) (5). irAEs can affect multiple organ systems, including the skin, endocrine glands, heart, lungs, gastrointestinal tract, and liver. These adverse events not only impair quality of life but may also lead to treatment interruption, which can be viewed as a form of functional resistance, making them a key concern in clinical management.

The importance of autoantibodies as potential predictive biomarkers of irAEs has increasingly been recognized. Clinically, autoantibodies, which are classic serological markers of autoimmune disease, may also be detected in some healthy individuals and in patients with subclinical autoimmune states (6, 7). Thus, patients without overt clinical manifestations or symptoms of autoimmune disease may still test positive for autoantibodies, reflecting a degree of immune susceptibility. Under the immune-stimulating pressure of ICIs, with or without concurrent chemotherapy, such susceptibility may accelerate the occult development of irAEs and further influence treatment tolerability and decisions regarding therapy discontinuation. This immune-susceptible state is not only closely associated with the risk of irAEs but also directly affects immunotherapy tolerance and treatment continuity. Importantly, the relationship between preexisting immune susceptibility and ICI outcomes is multifaceted: it may increase irAE risk, yet emerging evidence suggests it does not necessarily worsen irAE severity or compromise treatment continuity when properly managed.

In the study by Yoneshima et al. (8), the incidence of irAEs did not differ significantly between ANA-positive and ANA-negative patients (33.3% vs. 32.3%), and irAEs were mainly low grade (CTCAE grade 1–2). Notably, although PD-1 inhibitors could be administered safely in ANA-positive patients without obvious exacerbation of autoimmune disease, the presence of ANA was associated with significantly shorter progression-free and overall survival (p = 0.03 for both) [8]. This finding suggests that ANA positivity may indicate a poorer prognosis rather than simply increased irAE susceptibility, highlighting the need for careful risk stratification in this population. A prospective cohort study by Daban et al. (9) further confirmed that patients with preexisting autoantibodies were more likely to develop grade ≥2 irAEs (50% vs. 22%, OR = 3.5, P < 0.001), yet most could continue ICI therapy after standardized management. In addition, a case-control study by Isaacs et al. (10) showed that even in patients with a history of autoimmune disease, the incidence of severe irAEs was not significantly different from that in controls (21.4% vs. 16.1%, P = 0.627), and most irAEs resolved completely (66.7%).

Collectively, these findings suggest that preexisting autoimmunity or autoantibody positivity is associated with a higher frequency of irAEs but not necessarily with more severe or refractory toxicities. This observation raises an important question: if baseline immune susceptibility does not compromise tolerability, might it instead enhance antitumor immunity? Indeed, several studies have reported that patients who develop irAEs or who are autoantibody-positive may have better progression-free and overall survival (7, 11, 12), supporting the hypothesis that heightened baseline immune activation could potentiate antitumor responses. These two observations—increased irAE risk and potentially enhanced antitumor efficacy—are not mutually exclusive; rather, they may represent two facets of the same underlying immune hyper-reactivity. The net clinical outcome likely depends on the balance between these opposing effects, which may vary according to tumor type, treatment regimen, and individual immune status. However, the connection between autoantibody status, irAE patterns, and treatment outcomes—particularly the balance between increased irAE risk and potential therapeutic benefit—remains incompletely characterized in patients receiving ICI with or without concurrent chemotherapy.

Beyond predicting irAEs, baseline immune susceptibility may also influence therapeutic resistance, including potential modulation of chemosensitivity when ICIs are combined with chemotherapy —for instance, through enhanced immune-mediated tumor cell killing that could complement cytotoxic chemotherapy. Resistance to ICIs can be primary or acquired, with tumor-intrinsic mechanisms being well studied (3). However, host immune factors, including preexisting autoantibodies, may modulate treatment durability and the emergence of acquired resistance (11). Severe irAEs can force premature ICI discontinuation, leading to reduced treatment exposure and potentially suboptimal therapeutic effect. Conversely, heightened baseline immune activation might enhance antitumor immunity and delay acquired resistance, as suggested by the association between autoantibody positivity and improved progression-free survival (11, 12). Therefore, investigating preexisting autoantibodies in relation to irAE patterns and treatment continuity may provide insights into immune-mediated resistance and inform strategies to optimize treatment persistence.

From a biological perspective, the association between ANA positivity and irAE susceptibility may be underpinned by several interrelated mechanisms. Autoantibodies can reflect a state of underlying immune dysregulation characterized by reduced self-tolerance and enhanced T-cell reactivity, which may be amplified upon PD-1/PD-L1 blockade (4). In addition, preexisting autoantibodies may indicate a more inflamed tumor immune microenvironment, facilitating T-cell infiltration and activation that could augment both antitumor efficacy and off-target tissue damage (11). This dual effect—increased immune activity driving both tumor control and irAEs—provides a plausible biological basis for the observed associations. However, direct evidence linking ANA positivity to specific immune cell phenotypes or cytokine profiles in this context remains limited.

The present study aimed to characterize irAEs in a cohort of solid tumor patients who underwent autoantibody screening before ICI treatment and to further assess treatment tolerability and the possibility of continued ICI therapy under standardized management in ANA-positive patients. Given the retrospective design and limited sample size for subgroup analyses, this study is intended to be exploratory and hypothesis-generating rather than confirmatory.

## 2 Materials and methods

### 2.1 Study design and patients

We conducted a retrospective analysis at the China-Japan Union Hospital of Jilin University of patients with solid tumors who received single-agent or combination ICI therapy between August 2021 and February 2026. Patients with any systemic autoimmune disease were excluded. Retrospective data were extracted from the electronic medical record system and included clinical and demographic information, laboratory findings, imaging data, tumor characteristics, immune-related adverse events, and date of death or last follow-up.

All suspected irAEs, including dermatitis, thyroid dysfunction, hepatitis, hypophysitis, adrenal insufficiency, myocarditis, colitis, and interstitial pneumonitis, were assessed and graded according to the Common Terminology Criteria for Adverse Events (CTCAE) version 5.0.

The study was approved by the local institutional ethics review board.

Prior to ICI therapy, all included patients underwent comprehensive biological and physical examination. The inclusion criteria were: (1) pathologically confirmed solid tumor in patients aged ≥18 years, without an upper age limit; (2) treatment with anti-PD-1/PD-L1 monotherapy or combination therapy regardless of treatment line, disease stage (including metastatic disease), or treatment intent (including palliative settings); (3) The specific ICI agents administered included PD-1 inhibitors (sintilimab, tislelizumab, camrelizumab, toripalimab, nivolumab, pembrolizumab, serplulimab, penpulimab), PD-L1 inhibitors (sugemalimab, benmelstobart, adebrelimab), and bispecific antibodies (ivonescimab, cadonilimab); and (4) complete baseline autoantibody profile prior to treatment, including ANA, ENA, and thyroid autoantibodies, with a retrievable clinical indication for testing. There was no minimum requirement for the number of ICI cycles or treatment duration, as long as at least one dose of ICI was administered. In this study, “combination therapy” refers exclusively to ICI combined with chemotherapy or/and targeted therapy, as patients receiving dual immunotherapy (ICI plus ICI) were not included in sufficient numbers for meaningful analysis.

For the purposes of this study, patients were divided into two comparison groups based on baseline ANA titer: an ANA-positive group (titer ≥1:80) and an ANA-negative group (titer <1:80), as detailed in Section 2.2. These two groups were compared for the incidence, severity, organ distribution, onset time, glucocorticoid use, and immunotherapy outcomes of irAEs.

Because this was a retrospective study, autoantibody testing was not a systematic screening protocol predefined by the study but was performed according to individualized clinical judgment in routine practice (for example, baseline evaluation, suspected autoimmune background, or participation in a specific monitoring program). To minimize selection bias, we consecutively enrolled all patients meeting the above criteria with complete testing records. The two groups were well balanced in baseline characteristics (**Table 1**), providing a solid basis for subsequent comparative analyses, including subgroup analyses according to chemotherapy combination. Serum samples were collected to determine whether patients had one or more of the following preexisting autoantibodies.

**Table 1:** Baseline characteristics of patients stratified by ANA status.

Characteristic	ANA-positive group (n = 60)	ANA-negative group (n = 61)	P value
Age	64.5 (56.0–69.2)	65.0 (56.0–70.0)	0.926
Sex			0.444
Male	42 (70.0%)	38 (62.3%)	
Female	18 (30.0%)	23 (37.7%)	
BMI (kg/m²)	22.5 (20.9–24.5)	22.9 (21.4–25.6)	0.189
History of autoimmune disease	0 (0)	0 (0)	—
Baseline therapeutic corticosteroid use*	0 (0)	0 (0)	—
Smoking history			0.194
Yes	27 (45.0%)	20 (32.8%)	
No	33 (55.0%)	41 (67.2%)	
Alcohol use			0.519
Yes	15 (25.0%)	12 (19.7%)	
No	45 (75.0%)	49 (80.3%)	
Tumor type			0.899
Lung cancer	16 (26.7%)	18 (29.5%)	
Gastric cancer	15 (25.0%)	14 (23.0%)	
Esophageal cancer	8 (13.3%)	5 (8.2%)	
Hepatocellular carcinoma	3 (5.0%)	3 (4.9%)	
Others	18 (30.0%)	21 (34.4%)	
Tumor stage			0.220
Stage I	1 (1.7%)	2 (3.3%)	
Stage II	7 (11.9%)	2 (3.3%)	
Stage III	15 (25.4%)	12 (19.7%)	
Stage IV	36 (61.0%)	45 (73.8%)	
ICI type			0.842
PD-1 inhibitor	54 (90.0%)	55 (90.2%)	
PD-L1 inhibitor	3 (5.0%)	4 (6.6%)	
Bispecific immunotherapy	3 (5.0%)	2 (3.3%)	
Combined chemotherapy			1.000
Yes	48 (80.0%)	49 (80.3%)	
No	12 (20.0%)	12 (19.7%)	
Combined targeted therapy			0.212
Yes	12 (20.0%)	19 (31.1%)	
No	48 (80.0%)	42 (68.9%)	

*Baseline therapeutic corticosteroid use was defined as systemic corticosteroid administration for non-prophylactic indications within 7 days before the first ICI cycle (e.g., for symptom management of brain metastases, dyspnea, or autoimmune conditions). Single-dose prophylactic corticosteroids administered as premedication for chemotherapy were not counted as baseline corticosteroid use, as they were short-acting and not intended for immunosuppression. These premedications were balanced between the two groups in the combination chemotherapy subgroup.

### 2.2 Indirect immunofluorescence assay

According to fluorescence intensity at a serum dilution of 1:80, patients were divided into an ANA positive group (ANA+, titer ≥1:80) and an ANA negative group (ANA−, titer <1:80).The cutoff of 1:80 was chosen because titers between 1:40 and 1:80 are commonly considered low-positive and may be detected in healthy individuals without clinical significance (6, 7). Using a higher cutoff (≥1:80) reduces the likelihood of including non-specific, clinically irrelevant low-titer positivity and has been adopted in previous studies evaluating ANA as a predictor of irAEs (8, 13). This threshold thereby enhances the specificity of ANA positivity as a marker of clinically meaningful immune susceptibility in the context of ICI therapy.

### 2.3 Immunoblotting

Quantitative assessment of autoantibodies was performed, including ENA antibodies (U1nRNP, Sm, SSA, SSB, RO52, Scl70, PMScl, Jo1, CENPB), other nuclear antigens (dsDNA, PCNA, nucleosome, anti-histone, ribosomal P protein), mitochondrial antibody (AMAM2), and thyroid autoantibodies (anti-TPO, anti-TRAb).

### 2.4 Endpoints

The primary endpoint was the occurrence of irAEs. The study examined (1): clinical characteristics of irAEs, including onset time, severity, and occurrence of multiple toxicities; and (2) treatment tolerability and continuity, including initiation time of ICIs, whether irAEs resolved, time to resolution, management strategies (glucocorticoids or immunosuppressive agents), and whether treatment was discontinued because of irAEs or other reasons such as disease progression or patient preference.

### 2.5 Statistical analysis

Data was extracted from the electronic medical record system. Statistical analyses were performed using R software (version 4.3.1). Categorical variables (e.g., ANA status, incidence of irAEs, and severity grades) are presented as frequencies and percentages (%). Between-group differences were compared using the chi-square test or Fisher’s exact test. Odds ratios (ORs) and 95% confidence intervals (CIs) were calculated using Fisher’s exact test to quantify the strength of association.

For non-normally distributed continuous variables (such as time to onset of irAEs), the Mann–Whitney U test was used for group comparisons. For dose–response analysis of ANA titer levels (negative, 1:100, and 1:320), ANA titer was included as an ordinal variable in a logistic regression model to estimate the trend P value (P_trend) and assess the linear increase in risk with rising titer categories. Kaplan–Meier curves were used to plot the cumulative irAE-free proportion, and the log-rank test was used to assess differences in time-to-event distributions between groups. Finally, a multivariable Cox proportional hazards model was constructed to evaluate ANA status as an independent predictor of irAE occurrence after adjustment for age, sex, tumor stage, smoking history, alcohol use, BMI, combination chemotherapy and other clinical covariates. All statistical tests were two-sided, and P < 0.05 was considered statistically significant.

## 3 Results

### 3.1 Patient characteristics

A total of 121 patients were included. Based on the predefined ANA cutoff, 60 patients (49.6%) were classified as ANA positive (titer ≥1:80) and 61 (50.4%) as ANA negative (titer <1:80). Six patients (5.0%) had positivity for two or more autoantibodies. The ICIs used included PD-1 inhibitors, PD-L1 inhibitors, and bispecific immunotherapy agents. The ANA-positive and ANA-negative groups were highly balanced across all baseline characteristics. There were no significant differences in age (median 64.5 vs. 65.0 years), sex distribution (both predominantly male), BMI, smoking history, or alcohol consumption (all P > 0.05). As per the study exclusion criteria, no patient had a history of autoimmune disease. Furthermore, no patient received therapeutic systemic corticosteroids at baseline (defined as within 7 days before the first ICI cycle), although single-dose prophylactic premedication for chemotherapy was administered as clinically indicated and was balanced between groups. No statistically significant differences were observed in the major baseline variables, indicating excellent comparability between the two groups and providing a strong basis for evaluating the independent association between ANA status and adverse event risk. Nevertheless, residual confounding cannot be excluded; therefore, multivariable modeling was further applied. The clinical characteristics of the patients are summarized in **Table 1**.

### 3.2 Subgroup analysis and multivariable Cox regression

The overall incidence, severity, and organ distribution of irAEs are presented in detail in Sections 3.3, 3.6, and 3.7 below. To further evaluate the independent association between ANA status and irAE risk, a multivariable Cox proportional hazards regression model was constructed, using the time to first irAE occurrence as the event endpoint. The model included ANA status, age, sex, tumor stage (stage IV vs. I–III), ICI type (PD-1 vs. non–PD-1), tumor type, smoking history, alcohol use, BMI, and combination therapy as covariates.

After adjustment for age, sex, tumor stage, ICI type, and combination chemotherapy, the multivariable Cox model demonstrated that several factors were independently associated with irAE risk (**Table 2**). Female sex was significantly associated with an increased risk of irAEs (adjusted HR = 2.82, 95% CI: 1.70-4.68, P < 0.001). ANA positivity remained an independent predictor of irAEs (adjusted HR = 1.78, 95% CI: 1.09-2.91, P = 0.021). Notably, PD-1 inhibitor use was associated with a significantly reduced risk of irAEs compared with non-PD-1 inhibitors (adjusted HR = 0.31, 95% CI: 0.14-0.65, P = 0.002). Age (HR = 1.02, 95% CI: 0.99-1.05, P = 0.151), combination chemotherapy (HR = 1.58, 95% CI: 0.79-3.17, P = 0.193), and stage IV disease (HR = 0.68, 95% CI: 0.40-1.13, P = 0.138) were not significantly associated with irAE risk.

**Table 2:** Multivariable Cox regression analysis of factors associated with irAE risk.

Clinical variable	Hazard ratio (HR)	95% CI	P value
Sex (female vs. male)	2.82	1.70–4.68	<0.001
Age	1.02	0.99–1.05	0.151
ANA status (positive vs. negative)	1.78	1.09–2.91	0.021
Tumor stage (stage IV vs. I–III)	0.68	0.40–1.13	0.138
ICI type (PD-1 vs. non–PD-1)	0.31	0.14–0.65	0.002
Combination chemotherapy (yes vs. no)	1.58	0.79–3.17	0.193

### 3.3 Association between ANA status and irAE incidence

The incidence and severity of irAEs are summarized in this section, with organ-specific details provided in Section 3.6 and severity in Section 3.7. As shown in **Table 3** and **Figure 1**, ANA-positive patients had a significantly higher risk of irAEs than ANA negative patients. The incidence of irAEs in the ANA positive group was 70.0% (42/60), significantly higher than the 49.2% (30/61) observed in the ANA-negative group (χ² = 4.611, P = 0.032). Fisher’s exact test further confirmed this association (P = 0.026), showing that ANA-positive patients had a 2.41-fold higher risk of irAEs than ANA-negative patients (OR = 2.41, 95% CI: 1.14–5.09).

**Table 3:** Association between ANA status and irAE incidence.

Group	Total (N)	irAE	No irAE	Chi-square		P value (Chi-square)	OR (95% CI)	P value (Fisher)
ANA-negative	61	30 (49.2%)	31 (50.8%)		—	—	—	—
ANA-positive	60	42 (70.0%)	18 (30.0%)		4.611	0.032	2.41 (1.14–5.09)	0.026

**Figure 1:**
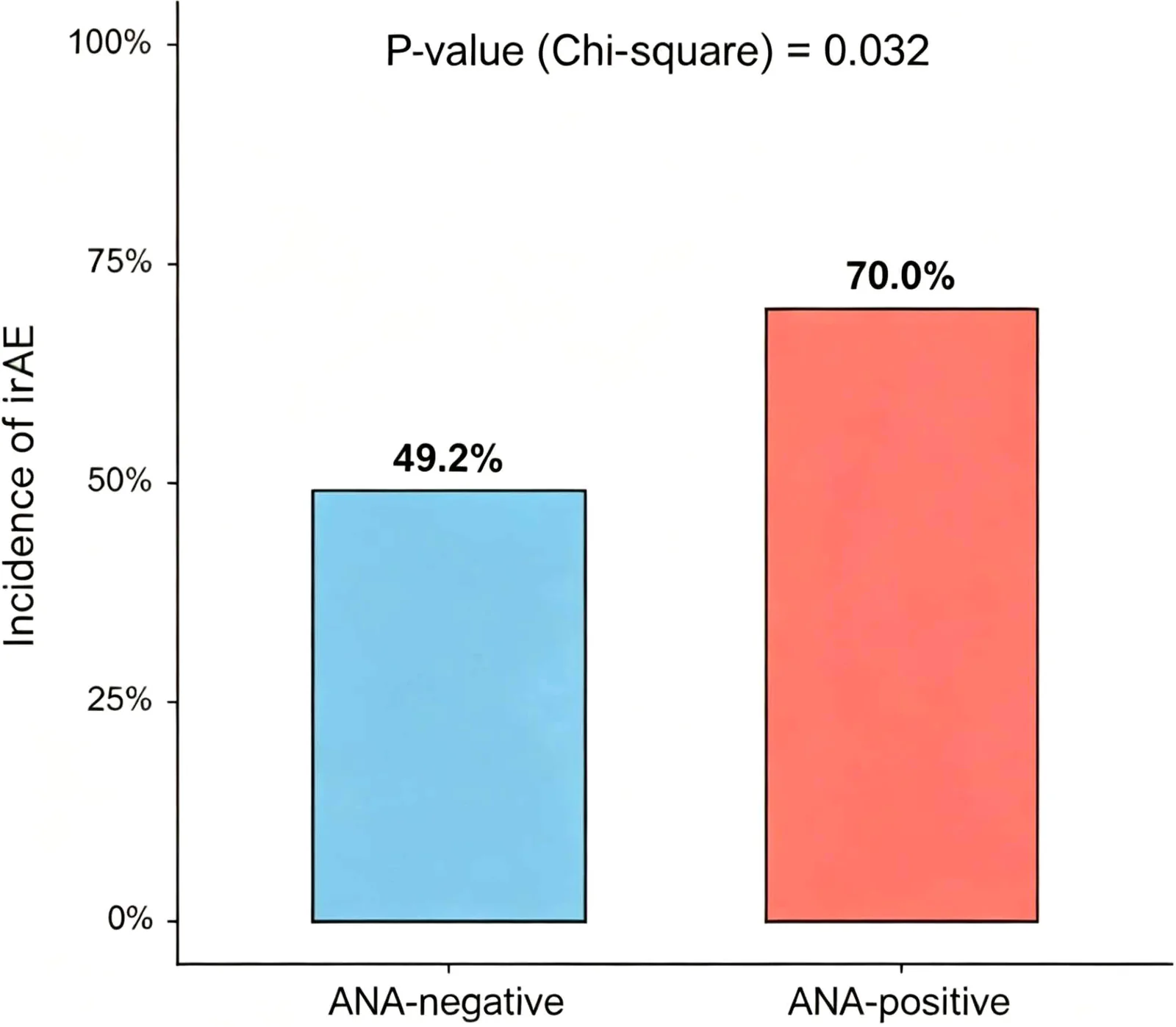
Comparison of irAE incidence between ANA-negative and ANA-positive patients.

### 3.4 Effect of ANA titer on irAE risk

To determine whether autoantibody titer had a dose effect relationship with irAE risk, we performed subgroup analysis across different titer levels (**Table 4**; **Figure 2**). Even low-titer positivity (1:100) was significantly associated with increased irAE risk (incidence 71.1%; compared with the ANA-negative group: OR = 2.54, 95% CI: 1.07–6.01, P = 0.038). When the titter increased to 1:320, the incidence remained high (70.6%); although the P value did not reach statistical significance because of the limited sample size (P = 0.170), the OR remained as high as 2.48.

**Table 4:** Statistical analysis of ANA titer and irAE incidence.

Comparison	irAE incidence in group 1 (n/N, %)	irAE incidence in group 2 (n/N, %)	OR (95% CI)	Chi-square	P value (Chi-square)	P value (Fisher)
1:100 vs. negative	27/38 (71.1%)	30/61 (49.2%)	2.54 (1.07–6.01)	3.734	0.053	0.038
1:320 vs. negative	12/17 (70.6%)	30/61 (49.2%)	2.48 (0.78–7.89)	1.666	0.197	0.170
1:320 vs. 1:100	12/17 (70.6%)	27/38 (71.1%)	0.98 (0.28–3.44)	0.000	1.000	1.000

**Figure 2:**
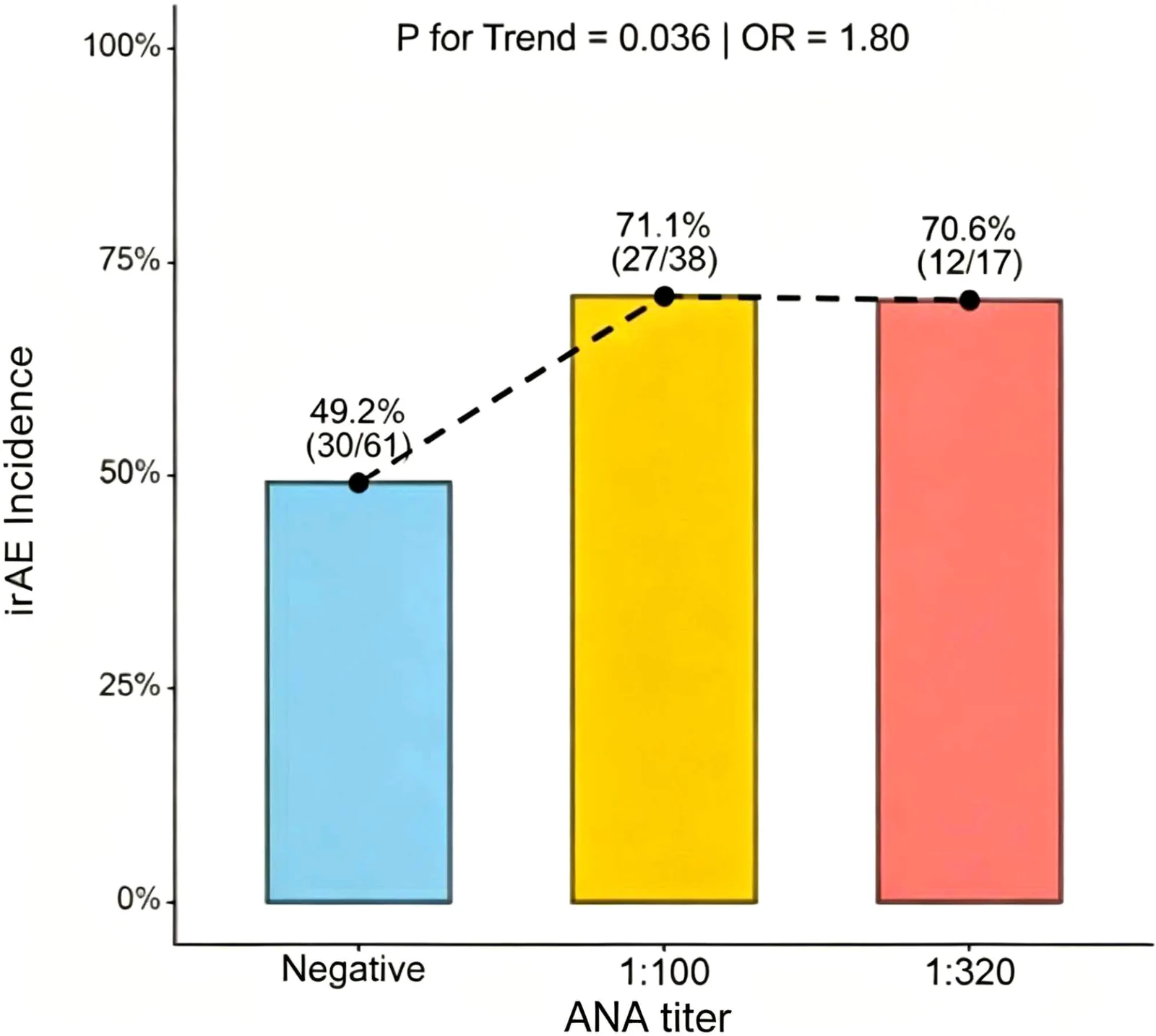
Comparison of irAE incidence across different ANA titer levels.

To further explore the effect of pre-treatment ANA titer on irAE risk, ANA titer was treated as an ordinal variable in a logistic regression trend test. The results showed a significant linear increase in irAE risk with rising ANA titer (P_trend = 0.036). However, the actual incidence rates did not show a progressive increase from the 1:100 to the 1:320 subgroup (71.1% vs. 70.6%), suggesting that the observed trend may be primarily driven by the difference between ANA-negative and any positive titer, rather than a strictly linear dose–response across all levels. The limited number of patients in the high-titer subgroup (n = 17) reduces the statistical power and precision of this estimate; therefore, the apparent plateau should be interpreted with caution. Logistic regression indicated that each increase in ANA titer category was associated with an 80% increase in irAE risk (OR = 1.80, 95% CI: 1.04–3.12), but this summary estimate relies on the ordinal model assumption and may not fully reflect the actual pattern. Taken together, these findings suggest an initial elevation in irAE risk at low-positive titers, with no clear further increase at higher titers in our sample; this pattern requires validation in larger cohorts with adequate numbers of high-titer patients before a definitive dose–response relationship can be established.

### 3.5 Effect of baseline autoantibody burden on irAE development

The incidence of irAEs increased with the number of positive autoantibodies present at baseline (**Supplementary Table 1**). In the autoantibody-negative group, the irAE incidence was 54.84%; in patients with one positive autoantibody, it increased to 72.73%; and in those with two or more positive autoantibodies, it reached 83.33%. Although the intergroup difference was not statistically significant in the current sample (P = 0.146), this dose–effect trend suggests that polyclonal immune activation at baseline, reflected by the presence of multiple autoantibodies, may indicate greater susceptibility to ICI-related immune toxicity.

Among the 72 patients who developed irAEs, we further examined the relationship between antibody burden and toxicity severity (**Supplementary Table 2**). The increase in the number of positive autoantibodies did not lead to more severe irAEs. The proportion of severe irAEs (grade ≥3) was 17.65% in the all-negative group, 18.75% in the one-positive group, and 0.00% in the ≥2-positive group, with no statistically significant difference between groups (P = 0.581). The mean maximum CTCAE grade also did not differ significantly among the groups (1.61, 1.56, and 1.20, respectively; P = 0.711).

### 3.6 Association between ANA positivity and organ-specific irAEs

As referenced in Sections 3.2 and 3.3 above, given the exploratory nature of organ-specific analyses and the limited sample size, the following findings should be interpreted as preliminary observations. Further analysis of organ-specific irAEs (**Table 5**; **Figure 3**) showed that ANA-positive patients had a trend toward higher risk in most organ systems except the kidney. Endocrine involvement was the most common, with an incidence of 50.0% in the ANA-positive group versus 36.1% in the ANA-negative group (OR = 1.77, P = 0.144). Pulmonary (10.0% vs. 4.9%, OR = 2.15) and hepatic (6.7% vs. 3.3%, OR = 2.11) involvement also showed more than a two-fold higher trend in the ANA-positive group. Cardiac (13.3% vs. 9.8%) and cutaneous (5.0% vs. 3.3%) irAEs were also slightly more frequent in the ANA-positive group, whereas renal involvement was rare. Although statistical significance was not reached for individual organ systems because of the limited sample size (all P > 0.05), this consistent increase across multiple organ systems aligns with the overall rise in irAE risk and suggests that ANA positivity may indicate a broader susceptibility to organ involvement. However, these subgroup comparisons are underpowered, and the observed patterns are exploratory; they should not be overinterpreted and require confirmation in larger, adequately powered studies.

**Table 5:** Statistical analysis of ANA positivity and organ-specific irAEs.

Organ system	Incidence in ANA-positive	Incidence in ANA-negative	Chi-square	P value (Chi-square)	OR (95% CI)	P value (Fisher)
Endocrine	30/60 (50.0%)	22/61 (36.1%)	1.862	0.172	1.77 (0.86–3.67)	0.144
Lung	6/60 (10.0%)	3/61 (4.9%)	0.517	0.472	2.15 (0.51–9.02)	0.323
Liver	4/60 (6.7%)	2/61 (3.3%)	0.193	0.660	2.11 (0.37–11.96)	0.439
Heart	8/60 (13.3%)	6/61 (9.8%)	0.101	0.751	1.41 (0.46–4.34)	0.583
Skin	3/60 (5.0%)	2/61 (3.3%)	0.000	0.985	1.55 (0.25–9.64)	0.680
Kidney	1/60 (1.7%)	2/61 (3.3%)	0.000	1.000	0.50 (0.04–5.67)	1.000

**Figure 3:**
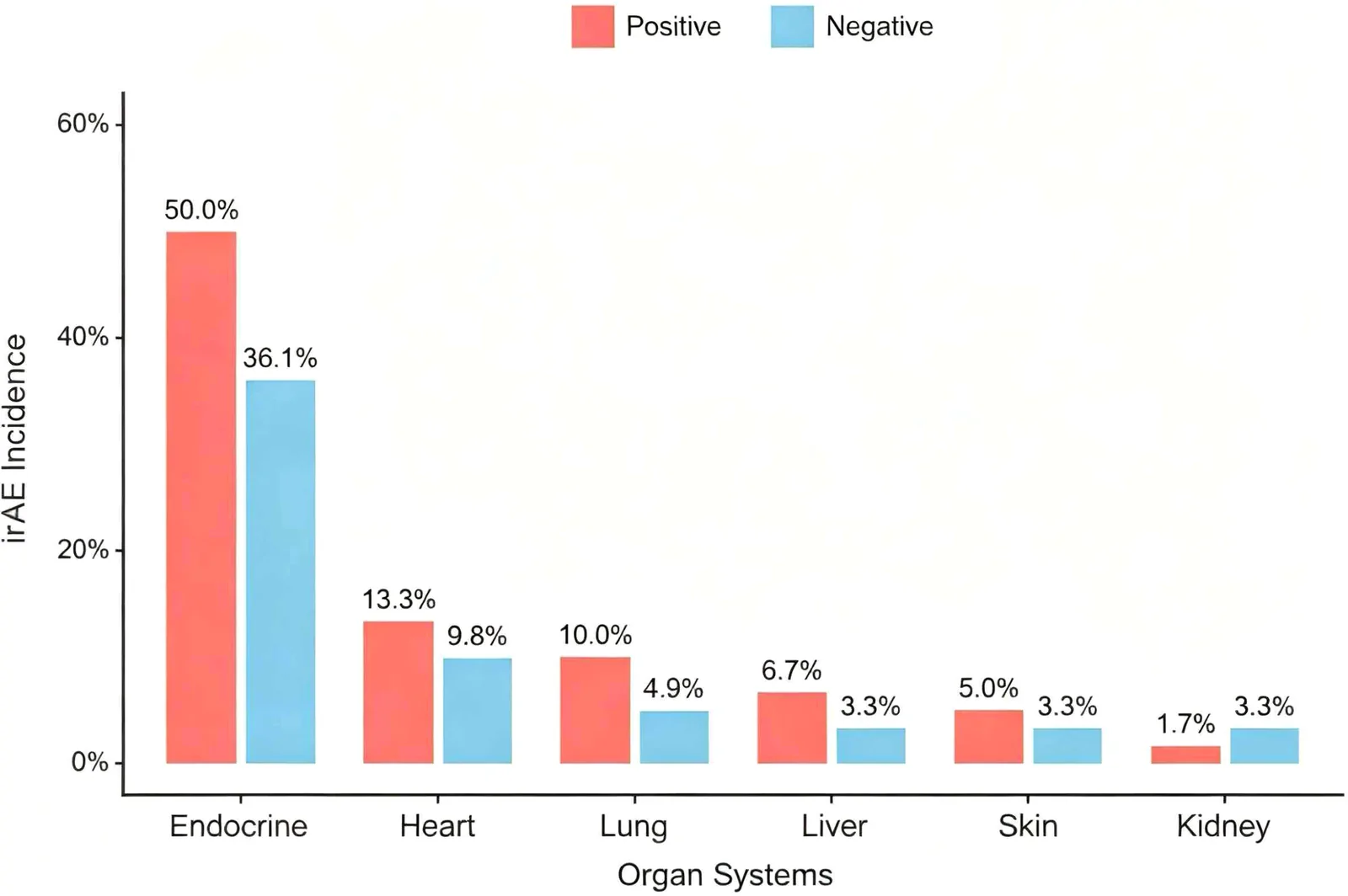
Comparison of organ-specific irAE incidence between ANA-negative and ANA-positive patients.

### 3.7 Association between ANA status and irAE severity

In terms of severity (**Table 6**; **Figure 4**), the proportion of grade ≥3 severe irAEs was 15.0% (9/60) in the ANA-positive group, higher than 8.2% (5/61) in the ANA-negative group. However, this difference did not reach statistical significance (P = 0.27). The corresponding OR was 1.98 (95% CI: 0.62–6.29), indicating a trend toward increased risk but with a wide confidence interval.

**Table 6:** Association between ANA status and irAE severity.

Group	Total (N)	Grade ≥3 irAE (n, %)	Chi-square	P value (Chi-square)	OR (95% CI)	P value (Fisher)
ANA-negative	61	5 (8.2%)	—	—	Reference	—
ANA-positive	60	9 (15.0%)	0.784	0.376	1.98 (0.62–6.29)	0.270

**Figure 4:**
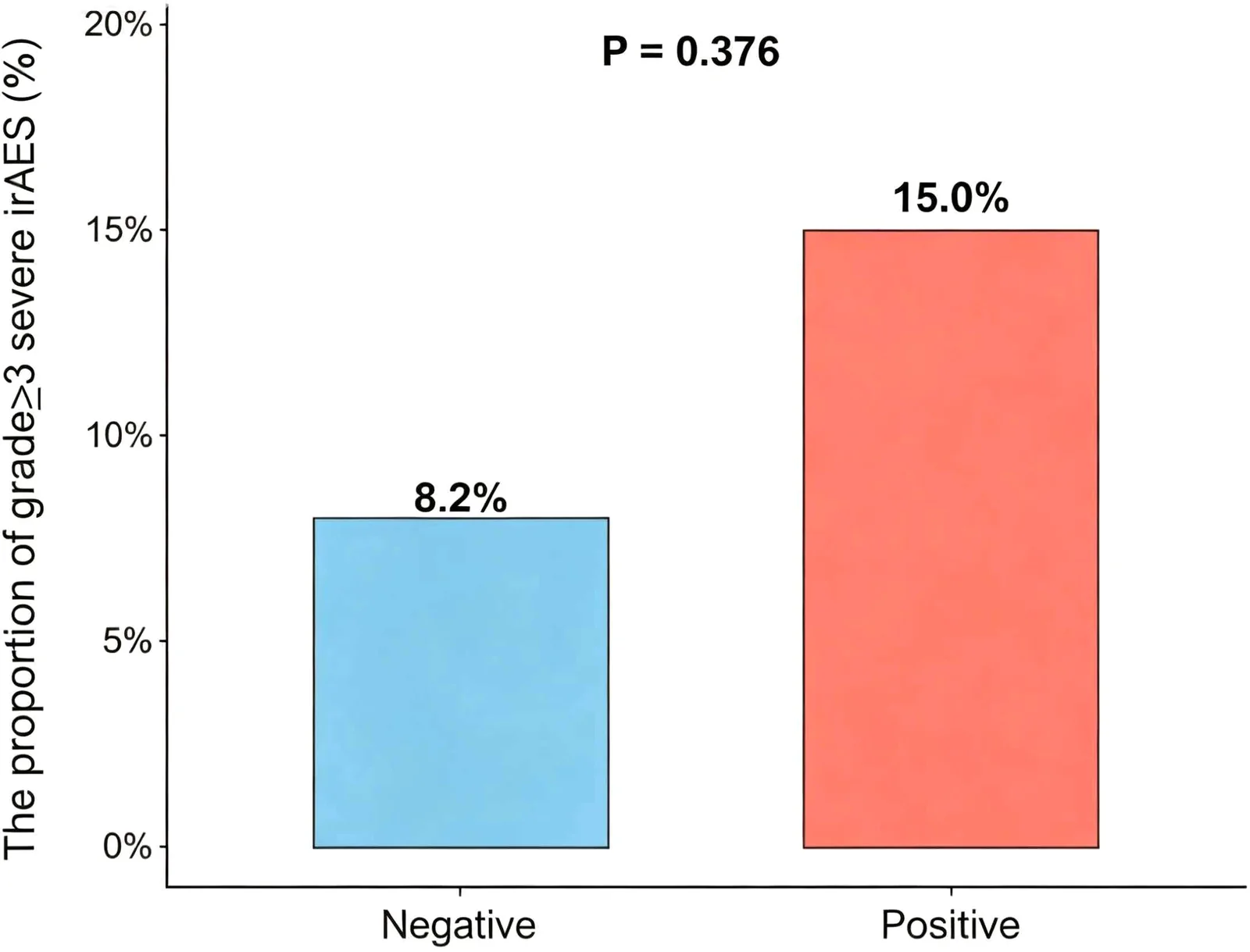
Comparison of severe irAE incidence (grade ≥3) between ANA-negative and ANA-positive patients.

### 3.8 Comparison of irAE onset time between ANA groups

Among patients who developed irAEs, we analyzed the time from immunotherapy initiation to onset. As shown in **Supplementary Table 3**, the median onset time in the ANA-positive group was 4.0 months (range: 0.7–28.0), which did not differ significantly from the 3.5 months (range: 0.4–13.4) observed in the ANA-negative group (P = 0.423). As shown in **Supplementary Table 4**, there was also no significant difference in onset time among ANA titer groups. These findings indicate that ANA status mainly affects whether irAEs occur, rather than changing the natural timing of onset.

### 3.9 Survival analysis

The effect of ANA status on cumulative irAE risk during treatment was assessed using Kaplan–Meier curves of irAE-free survival. As shown in **Figure 5**, the irAE-free proportion decreased more rapidly in the ANA-positive group over time, and the two curves separated clearly. The log-rank test confirmed that the probability of remaining irAE-free during follow-up was significantly lower in the ANA-positive group than in the ANA-negative group (P = 0.031).

**Figure 5:**
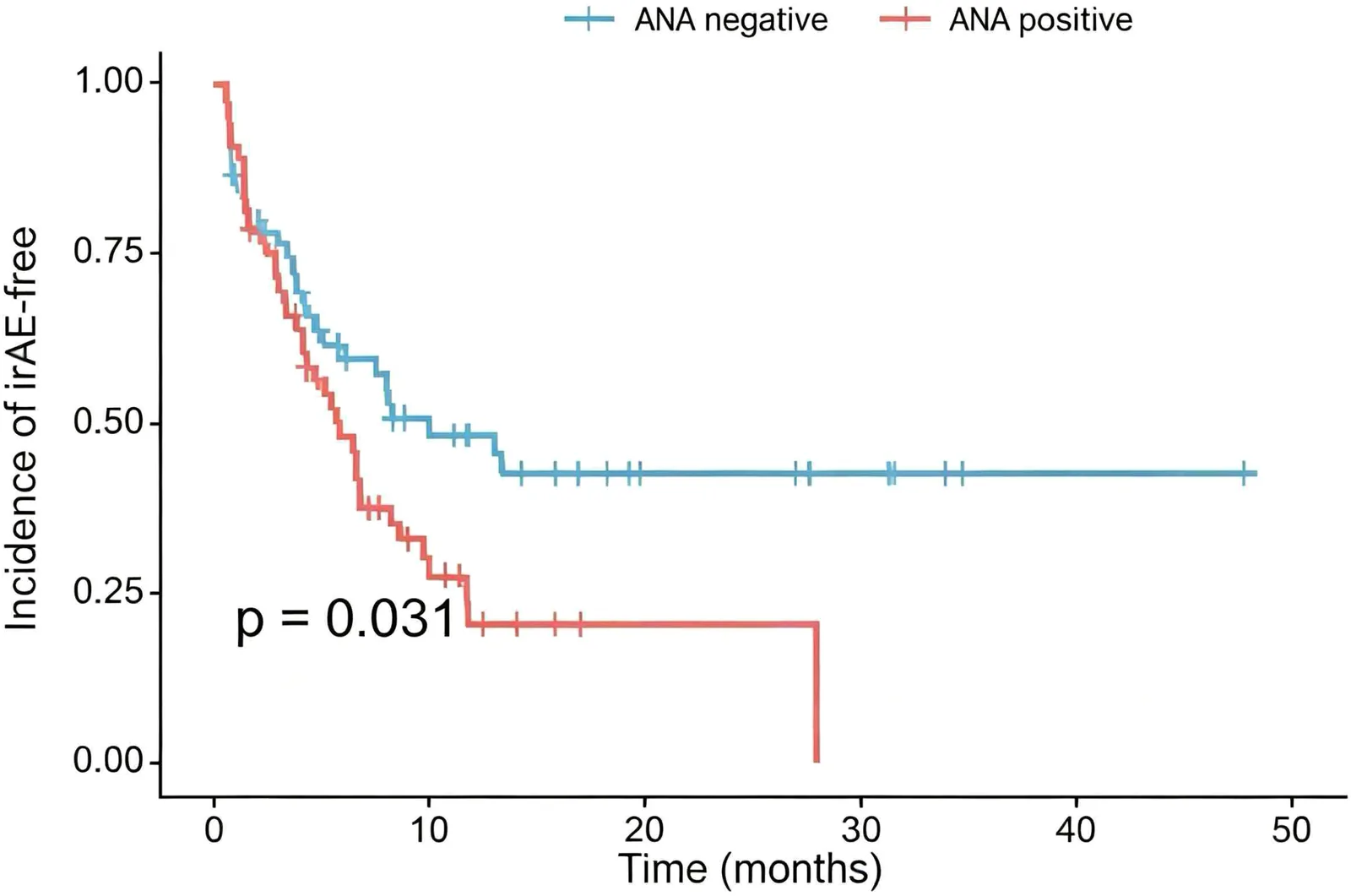
Kaplan–Meier curves of irAE-free survival according to ANA status.

We further conducted survival stratification by ANA titer (negative, 1:100, and 1:320) (**Supplementary Figure 1**). The Kaplan–Meier curves showed a more rapid decline in irAE-free proportion in both the low-titer (1:100) and high-titer (1:320) groups compared with the negative group. The overall log-rank test showed a near-significant difference among the groups (P = 0.071).

### 3.10 Immunotherapy outcomes

Analysis of immunotherapy outcomes (**Table 7**) showed that ANA status did not negatively affect overall treatment tolerability. The proportions of patients who discontinued treatment because of irAEs (20.0% vs. 21.3%, P = 1.000) and those who remained on treatment (21.7% vs. 23.0%, P = 1.000) were essentially the same in the two groups. Notably, the reasons for treatment discontinuation showed a numerical shift between groups: the proportion of discontinuation due to disease progression was lower in the ANA-positive group than in the ANA-negative group (21.6% vs. 34.4%, OR = 0.53, P = 0.157), whereas the proportion of discontinuation for other reasons was correspondingly higher (36.7% vs. 21.3%, OR = 2.12, P = 0.073). Details of the “other reasons” category are provided in the **Table 7** footnote and included patient preference, transition to hospice/palliative care, and loss to follow-up.Although these numerical differences might suggest that ANA-positive patients tolerate ICI-related toxicities adequately, we cannot draw firm conclusions about treatment tolerability or management efficacy from these data alone. Detailed data on specific management strategies, severity mitigation, supportive care measures, or irAE recovery outcomes were not systematically captured in this retrospective cohort.

**Table 7:** Association between ANA status and immunotherapy outcomes.

Outcome	ANA-positive (n/N, %)	ANA-negative (n/N, %)	OR (95% CI)	P value
Discontinued because of irAE	12/60 (20.0%)	13/61 (21.3%)	0.92 (0.38–2.23)	1.000
Discontinued because of progression	13/60 (21.6%)	21/61 (34.4%)	0.53 (0.21–1.27)	0.157
Still receiving treatment	13/60 (21.7%)	14/61 (23.0%)	0.93 (0.36–2.39)	1.000
Discontinued for other reasons	22/60 (36.7%)	13/61 (21.3%)	2.12 (0.89–5.24)	0.073

This shift suggests a potential trend that ANA-positive patients may tolerate ICI-related toxicities well, and that ANA positivity may reflect stronger antitumor immune activation, potentially reducing the risk of treatment termination due to disease progression, which is consistent with a delayed onset of acquired resistance to ICIs and/or chemotherapy. However, as these differences did not reach statistical significance, the hypothesis that ANA positivity delays acquired resistance to ICIs and/or chemotherapy remains unproven and requires further validation in larger prospective studies.

Among patients who developed irAEs, we further analyzed the association between ANA status and glucocorticoid use (**Table 8**). The proportion of ANA-positive patients receiving glucocorticoids for irAEs was 33.3% (14/42), numerically higher than that in ANA-negative patients (23.3%, 7/30). However, the chi-square test showed no statistically significant difference between the groups (χ² = 0.847, P = 0.357). In addition, the increased risk of corticosteroid use associated with ANA positivity did not reach statistical significance (OR = 1.64, 95% CI: 0.57–4.75). Taken together, these results indicate that although ANA-positive patients are more likely to develop systemic irAEs, these adverse events are usually within the range of routine clinical management and do not significantly increase the need for steroid therapy.

**Table 8:** Association between ANA status and glucocorticoid use among patients with irAEs.

ANA status	Steroid use (n = 21)	No steroid use (n = 51)	Total (N = 72)	Steroid use rate (%)	Chi-square	P value (Chi-square)	OR (95% CI)	P value (Fisher)
ANA-negative	7	23	30	23.3%	—	—	Reference	—
ANA-positive	14	28	42	33.3%	0.847	0.357	1.64 (0.57–4.75)	0.436

## 4 Discussion

In this single-center retrospective study, we evaluated the clinical characteristics and treatment outcomes of immune-related adverse events in patients with malignancies who were positive for antinuclear antibodies and received immune checkpoint inhibitor therapy with or without chemotherapy, and explored their implications for drug resistance.

Our findings provide robust multidimensional evidence supporting the predictive value of ANA as a biomarker for irAEs. The incidence of irAEs was markedly higher in ANA-positive patients than in ANA-negative patients, and this association remained robust after multivariable adjustment (adjusted HR = 1.78, P = 0.021). Notably, the effect size in our cohort aligns closely with that observed by Ceccarelli et al. (RR = 2.01) (7), further supporting the predictive role of ANA.

Second, our study revealed a significant positive trend across ANA titer categories (P_trend = 0.036), which is consistent with the observation by Hsu et al. in patients with liver cancer (13). Kohno et al. also confirmed in a large cohort of 626 patients that elevated baseline ANA titers (≥1:40) were significantly associated with the risk of grade ≥2 irAEs (14). However, the actual incidence rates did not show a progressive increase from low-positive (1:100) to high-positive (1:320) titers (71.1% vs. 70.6%), suggesting that the risk elevation is primarily driven by the presence of any positive titer rather than a strictly linear dose–response. The high-titer subgroup was small (n = 17), limiting statistical power; thus, the apparent plateau should be interpreted cautiously and requires validation in larger cohorts.

Regarding organ-specific clinical characteristics of irAEs, the ANA-positive group showed higher incidence trends in several organ systems, including endocrine (50.0% vs. 36.1%), pulmonary (10.0% vs. 4.9%), and hepatic (6.7% vs. 3.3%) toxicities, although none of the individual organ comparisons reached statistical significance. Given the exploratory nature and limited sample size, these organ-specific findings are hypothesis-generating rather than confirmatory. The consistent increase across multiple organs suggests a generalized immune susceptibility rather than organ-targeted autoimmunity, a notion supported by Daban et al. (9), who reported higher multisystem toxicity in autoantibody-positive patients.

We also analyzed the effect of baseline autoantibody burden. Among patients carrying multiple autoantibodies, irAE incidence increased progressively (0 positive: 54.84%; 1 positive: 72.73%; ≥2 positive: 83.33%), although the differences did not reach statistical significance (P = 0.146). This dose–effect trend suggests that polyclonal immune activation may indicate greater susceptibility to irAEs. Notably, increasing autoantibody burden did not lead to more severe irAEs: the severe irAE rate in the ≥2-positive group was 0%, and the mean CTCAE grade was only 1.20, lower than the 1.61 observed in the all-negative group (P = 0.711). This finding further supports the clinical pattern of high frequency but low grade, meaning that patients with a stronger baseline immune activation state may be more likely to develop irAEs, yet their toxicity tends to be milder. Together, these results suggest that baseline autoimmune status, whether subclinical or overt, does not necessarily translate into refractory clinical outcomes, and may even offer an opportunity to overcome resistance through enhanced immune activation.

A novel aspect of our study is the positive impact of ANA positivity on immunotherapy outcomes.Ceccarelli et al. similarly reported that patients who developed irAEs had significantly longer progression-free survival than those who did not (P = 0.007) (7), which is consistent with the association observed in our study between irAEs and treatment benefit. Giannicola et al. further showed that in non-small cell lung cancer patients treated with nivolumab, early detection of ANA and ENA autoantibodies was significantly associated with irAE risk (P = 0.002) and with longer progression-free survival (HR = 0.23, P = 0.004) and overall survival (HR = 0.28, P = 0.03) (12). Although ANA-positive patients had a higher incidence of irAEs, the proportion of treatment discontinuation due to irAEs was comparable between groups (20.0% vs. 21.3%). Although, these differences did not reach statistical significance (both P > 0.05), this numerical trend might suggest that ANA-positive patients tolerate ICI therapy well, and possibly exhibit stronger antitumor immune activation, but we cannot draw firm conclusions about therapeutic benefit or delayed resistance from these data alone. Importantly, we did not analyze progression-free survival (PFS) or overall survival (OS) in this cohort; thus, all references to survival benefit or delayed acquired resistance are speculative and derived from published literature rather than from original data from this study (11, 12). This shift in the distribution of treatment-ending events strongly suggests that, under standardized management, ANA-positive patients not only tolerate ICI therapy well but may also exhibit stronger antitumor immune activation, thereby prolonging therapeutic benefit.

From a drug resistance perspective, including chemoresistance when ICIs are combined with chemotherapy, severe irAEs can force premature ICI discontinuation, creating a state of “functional resistance” where therapy is interrupted before its full effect (5). Conversely, the reduced progression-driven discontinuation in ANA-positive patients raises the hypothesis that baseline autoimmunity might delay acquired resistance to ICIs and potentially to chemotherapy, consistent with reports that autoantibody positivity is associated with longer progression-free and overall survival (13, 14). Although we lack direct tumor immune microenvironment (TIME) data, our findings raise the hypothesis that ANA positivity could reshape TIME toward a more inflamed state, counteracting resistance mechanisms such as T cell exhaustion (11)—a process that may be relevant to both immunotherapy and chemotherapy response.

Importantly, in our study, the use of glucocorticoids for irAEs and the incidence of grade ≥3 severe irAEs were comparable between the two groups, with no statistically significant differences. These results together indicate that irAEs in ANA-positive patients are more frequent but are generally manageable and do not require stronger immunosuppressive intervention. This pattern is consistent with the observations of Yoneshima et al. in non-small cell lung cancer, where ANA-positive patients mainly experienced low-grade (grade 1–2) irAEs, and treatment discontinuation due to irAEs was not significantly different from that in ANA-negative patients (8). Similarly, Toi et al. reported that although patients with preexisting autoantibodies were more likely to develop irAEs (OR = 3.25, P = 0.001), patients with preexisting antibodies who developed irAEs could often continue ICI therapy, and both progression-free survival and overall survival were significantly improved (HR = 0.53, P = 0.002) (11).

Alserawan et al., in a prospective study of 152 patients with solid tumors, reported that treatment-emergent ANA changes were significantly associated with severe irAEs (83.3% of patients with severe irAEs showed altered ANA patterns), whereas baseline ANA status was not significantly associated with overall irAE incidence (15). This apparent discrepancy may be related to differences in testing time points and suggests that dynamic monitoring of autoantibody changes may be more predictive than a single baseline measurement. In patients with melanoma, Lue et al. found that baseline autoantibody profiles could predict irAEs, identifying 47 autoantibodies associated with irAEs, among which KRT7, RPLP2, UBE2Z, and GPHN were the strongest predictors, with predictive profiles differing according to ICI type (PD-1 monotherapy vs. CTLA-4 monotherapy vs. combination therapy) (16). This suggests that the predictive value of autoantibodies may differ depending on cancer type and immunotherapy regimen. Zhang et al. reported that baseline ANA positivity was not significantly associated with overall irAE incidence, though associations with specific toxicity patterns may exist (17). Izawa et al., in a study of 275 Japanese patients with solid tumors, also found no significant association between baseline ANA and overall irAE incidence, but baseline thyroid autoantibody positivity significantly predicted thyroid dysfunction (P < 0.01) (18). These conflicting results may be due to differences in ethnicity, ANA cutoff values (1:40 vs. 1:80), irAE grading criteria, and tumor composition, and they also suggest that the predictive value of autoantibodies may be cancer-specific and ethnicity-specific (14). Mu-Mosley et al. further showed that changes in autoantibody profiles are closely related to ICI type: regimens containing ipilimumab often increase autoantibody levels, whereas nivolumab monotherapy tends to be associated with decreases (19). Together, these findings indicate that future studies should integrate tumor type, treatment regimen, testing time point, and ethnic background to more accurately evaluate the predictive value of autoantibodies.

This study has several limitations. First, ANA testing was not performed as part of a prespecified systematic screening protocol for all patients, but was ordered based on individual clinical judgment (e.g., baseline evaluation, suspicion of underlying autoimmunity, or anticipated irAE risk). This introduces potential confounding by indication, as clinicians may have selectively tested patients perceived to have higher immune susceptibility. Although measured baseline characteristics were well balanced between groups (**Table 1**), residual confounding from unmeasured factors—such as the specific reasons for ANA testing, occult autoimmune conditions, or undocumented corticosteroid exposure—cannot be fully excluded. This selection bias should be considered when interpreting the association between ANA positivity and irAE risk. Second, we did not provide detailed information on the specific ICI agents used. Different PD-1/PD-L1 inhibitors and combination regimens (e.g., with chemotherapy or targeted therapy) carry differing risks and patterns of irAEs, particularly for organ-specific toxicities. Without specifying the exact agents, we cannot exclude treatment-related confounding, especially when interpreting organ-specific findings. Future studies should stratify by ICI type and combination therapy to clarify these associations. Our observation of reduced progression-driven discontinuation in ANA-positive patients needs validation in larger cohorts with progression-free survival analysis. Finally, given the expanding view of cancer resistance to include host immune networks, we propose baseline autoantibody testing as a stratification factor in future trials for both efficacy and resistance endpoints (3, 5).

## 5 Conclusion

Baseline ANA positivity is associated with a higher risk of irAEs after ICI therapy in patients with solid tumors and demonstrates a clear dose–response relationship. Although ANA-positive patients are more likely to develop irAEs, these events are generally manageable and do not appear to compromise treatment continuity. Baseline autoantibody testing may help identify patients at increased risk for irAEs and support individualized resistance-overcoming monitoring strategies during immunotherapy; however, its role in predicting or overcoming resistance is not established by our data and requires further investigation in studies with survival endpoints.

## Data Availability

The original contributions presented in the study are included in the article/**Supplementary Material**. Due to privacy‑related ethical restrictions, raw data cannot be publicly deposited; further inquiries can be directed to the corresponding authors.
