# Supplementary material for: Clinical characteristics of immune-related adverse events after immune checkpoint inhibitor therapy with or without chemotherapy in patients with antinuclear antibody-positive malignancies
Source: Front Immunol. 2026 Sep 14;17:1854574. doi: 10.3389/fimmu.2026.1854574 (PMC13616661; doi:10.3389/fimmu.2026.1854574)
Supplement: Supplementary file 1 [file Table1.docx]

**Supplementary files**

| **Supplementary**  **appendix** | **Page** |
| --- | --- |
| **Supplementary table S1. Association Between Baseline Autoantibody Burden and irAE Risk (All Patients)** | **1** |
| **Supplementary table S2. Association Between Baseline Autoantibody Burden and irAE Severity (Patients With irAEs)** | **2** |
| **Supplementary table S3.Comparison of irAE Onset Time Between ANA Groups** | **3** |
| **Supplementary table S4. Comparison of irAE Onset Time by ANA Titer** | **4** |
| **Supplementary figure S1. Kaplan–Meier curves of irAE-free survival according to ANA titer** | **5** |

**Supplementary Table S1. Association Between Baseline Autoantibody Burden and irAE Risk (All Patients)**

| Number of abnormal autoantibodies | Total patients | Patients with irAE | irAE incidence (%) | P value (chi-square test) |
| --- | --- | --- | --- | --- |
| 0 positive (all negative) | 93 | 51 | 54.84% | 0.146 |
| 1 positive | 22 | 16 | 72.73% |  |
| ≥2 positive | 6 | 5 | 83.33% |  |

**Supplementary Table S2. Association Between Baseline Autoantibody Burden and irAE Severity (Patients With irAEs)**

| Number of abnormal autoantibodies | Total with irAE (N = 72) | Severe irAE (grade ≥3) | Severe incidence (%) | Mean CTCAE grade | P value (chi-square test) |
| --- | --- | --- | --- | --- | --- |
| 0 positive (all negative) | 51 | 9 | 17.65% | 1.61 | 0.581 |
| 1 positive | 16 | 3 | 18.75% | 1.56 |  |
| ≥2 positive | 5 | 0 | 0.00% | 1.20 |  |

**Supplementary Table S3. Comparison of irAE Onset Time Between ANA Groups**

| Group | Total (N) | irAE events (n) | Median onset time (months) | Mean onset time (months) | Range (months) | P value (Mann–Whitney) |
| --- | --- | --- | --- | --- | --- | --- |
| ANA-negative | 61 | 30 | 3.5 | 4.1 | 0.4–13.4 | — |
| ANA-positive | 60 | 42 | 4.0 | 5.0 | 0.7–28.0 | 0.423 |

**Supplementary Table S4. Comparison of irAE Onset Time by ANA Titer**

| Group | Total (N) | irAE events (n) | Median onset time (months) | Mean onset time (months) | Range (months) | P value (vs. negative) |
| --- | --- | --- | --- | --- | --- | --- |
| Negative | 61 | 30 | 3.5 | 4.1 | 0.4–13.4 | — |
| 1:100 | 38 | 27 | 4.2 | 5.4 | 0.1–28.0 | 0.452 |
| 1:320 | 17 | 12 | 2.9 | 3.8 | 0.7–11.8 | 0.944 |

**Supplementary Figure S1. Kaplan–Meier curves of irAE-free survival according to ANA titer**
